# Pre-Columbian Floristic Legacies in Modern Homegardens of Central Amazonia

**DOI:** 10.1371/journal.pone.0127067

**Published:** 2015-06-01

**Authors:** Juliana Lins, Helena P. Lima, Fabricio B. Baccaro, Valdely F. Kinupp, Glenn H. Shepard, Charles R. Clement

**Affiliations:** 1 Programa de Pós-Graduação em Botânica, Instituto Nacional de Pesquisas da Amazônia (INPA), Manaus, Amazonas, Brasil; 2 Coordenação de Ciências Humanas, Museu Paraense Emílio Goeldi (MPEG), Belém, Pará, Brasil; 3 Departamento de Biologia, Universidade Federal do Amazonas (UFAM), Manaus, Amazonas, Brasil; 4 Herbário EAFM, Instituto Federal de Educação, Ciência e Tecnologia do Amazonas (IFAM), Manaus, Amazonas, Brasil; 5 Departamento de Antropologia, Museu Paraense Emilio Goeldi (MPEG), Belém, Pará, Brasil; 6 Coordenação de Tecnologia e Inovação, Instituto Nacional de Pesquisas da Amazônia (INPA), Manaus, Amazonas, Brasil; University of Oxford, UNITED KINGDOM

## Abstract

Historical ecologists have demonstrated legacy effects in apparently wild landscapes in Europe, North America, Mesoamerica, Amazonia, Africa and Oceania. People live and farm in archaeological sites today in many parts of the world, but nobody has looked for the legacies of past human occupations in the most dynamic areas in these sites: homegardens. Here we show that the useful flora of modern homegardens is partially a legacy of pre-Columbian occupations in Central Amazonia: the more complex the archaeological context, the more variable the floristic composition of useful native plants in homegardens cultivated there today. Species diversity was 10% higher in homegardens situated in multi-occupational archaeological contexts compared with homegardens situated in single-occupational ones. Species heterogeneity (β-diversity) among archaeological contexts was similar for the whole set of species, but markedly different when only native Amazonian species were included, suggesting the influence of pre-conquest indigenous occupations on current homegarden species composition. Our findings show that the legacy of pre-Columbian occupations is visible in the most dynamic of all agroecosystems, adding another dimension to the human footprint in the Amazonian landscape.

## Introduction

Humans have domesticated landscapes in varying degrees [1, 2] at an accelerating pace as population expanded during the Holocene and into the Anthropocene [3]. Studies in archaeology have identified long-term patterns, especially of plant and animal domestications [4, 5] that represent the legacies of long forgotten peoples. An increasing number of historical ecologists are identifying biodiversity legacies of long-term landscape domestication near ancient settlements, especially in forests in Europe [6], Mesoamerica [7], Africa [8] and Oceania [9]. Forests that were managed in the past may exhibit different legacies in the present depending on their varying occupation histories, often pre-historic [10], and their distance from archaeological sites [6]. In Amazonia some forests contain archaeological artifacts, including crop phytoliths, but many do not [11], suggesting that those forests were never cleared for food production [1]. However, floristic compositions found in modern anthropogenic [12] and mature forests [13] may be consequences of a mosaic of management systems, and these signals can be identified in the present by ecological and ethnobotanical methods [14]. The aim of this paper is to use these methods to identify legacy effects where nobody has ever looked: modern homegardens in traditional communities.

Homegardens are agroforestry systems and their structure includes several strata combining domesticated crops and wild plants with multiple uses. Their floristic composition is attributed to a multitude of factors, such as available area, garden age, soil quality, soil seed bank, surrounding vegetation and personal preferences [15]. They are found throughout the world where people have enough space, especially in the tropics. In Amazonia, traditional communities and their homegardens are commonly found in archaeological sites that contain *terra preta de índio* (TPI; Amazonian dark earth); within communities homegardens on TPI often have greater species diversity compared to homegardens on other soils [16, 17]. TPI are highly fertile anthropogenic soils rich in phosphorous, calcium, magnesium, charcoal and indigenous ceramics, created by the daily household activities of pre-Columbian societies between 2,500 and 500 years ago [18]. They are extremely abundant in many parts of Amazonia [19], especially on bluffs along major and tributary rivers, locations that are also preferred by modern communities [20]. The stylistic and technological traits of pre-Columbian ceramics, and the stratigraphy of these sites provide information about past cultures, and are used by archaeologists to map regional traditions and their changes through time. Archaeological sites may contain one or more ceramic traditions in different soil layers that generally represent distinct occupations [21]. During European conquest and subsequent colonization most of these sites were abandoned, and many were re-occupied later by modern Brazilians because of their rich soils and favorable locations.

**Fig 1:**
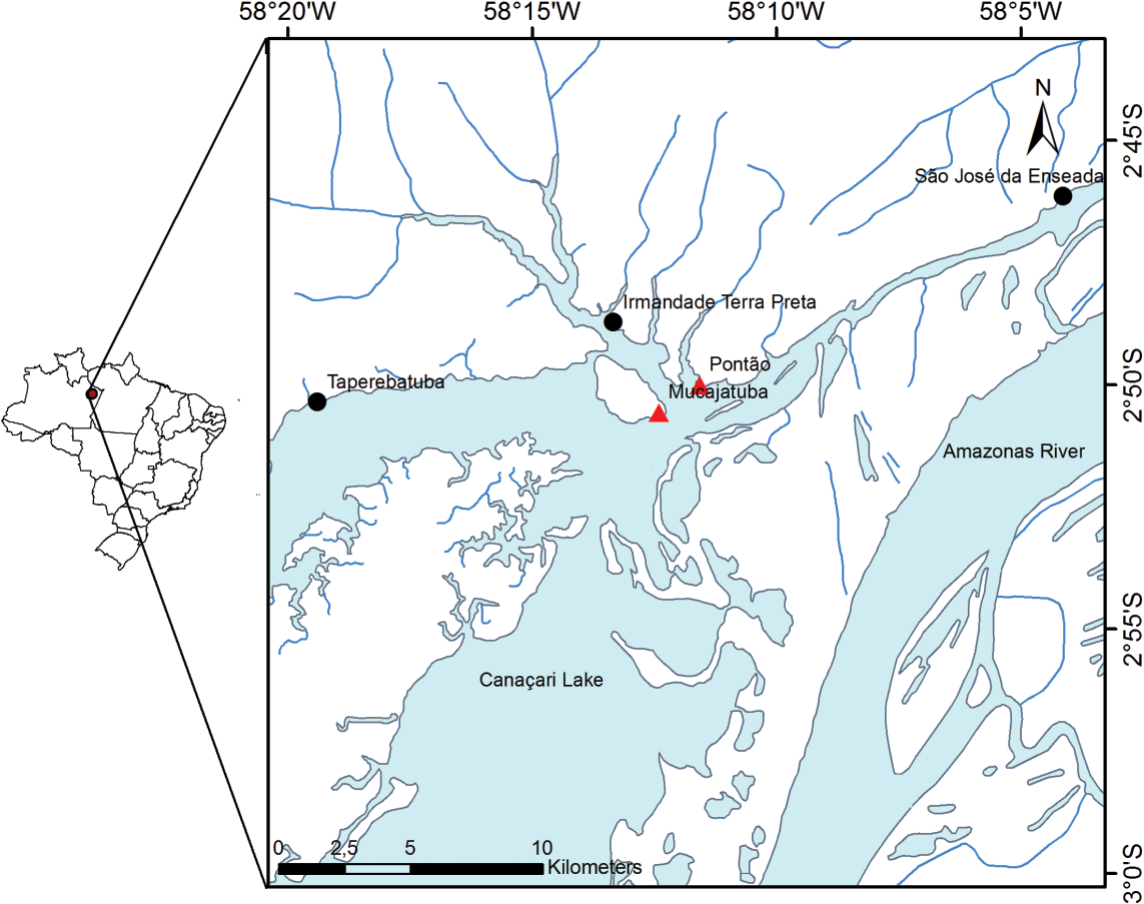
Study area. The big river entering from the left is the Urubu River that meets Lake Canaçari in front of Silves Island and flows into the Amazon River. The circles represent the communities with complex pre-Columbian occupational histories and triangles represent communities with simple pre-Columbian occupational histories, all of them along the Urubu River, in the State of Amazonas, Brazil, except for the Irmandade Terra Preta, which is near the mouth of the Itapani River, the last major tributary of the Urubu River.

We studied the modern floristic composition of 40 homegardens of traditional non-indigenous communities. These communities are situated on five archaeological sites containing TPI along the Urubu River, a northern tributary of the Amazon River, 200 km east of Manaus, Amazonas, Brazil (Fig 1). All study sites were reoccupied during the last 100–150 years by Brazilian smallholder farmers. Hence, every site has a modern occupation and one or more pre-Columbian occupations. We carried out inventories in homegardens to ascertain whether floristic composition varied between simple (single pre-Columbian occupation) and complex (more than one pre-Columbian occupation) archaeological contexts. Floristic composition was compared both for useful species native to Amazonia, which are more directly related to the indigenous past, and the whole set of useful species, including those introduced from other regions of the Americas and the Old World. In order to test the null hypothesis that there was no correlation between present plant composition and pre-Columbian indigenous management, we measured β-diversity for each archeological context and controlled the effects of multiple variables driving homegarden floristic variability using mixed effects models [22].

## Results and Discussion

**Fig 2:**
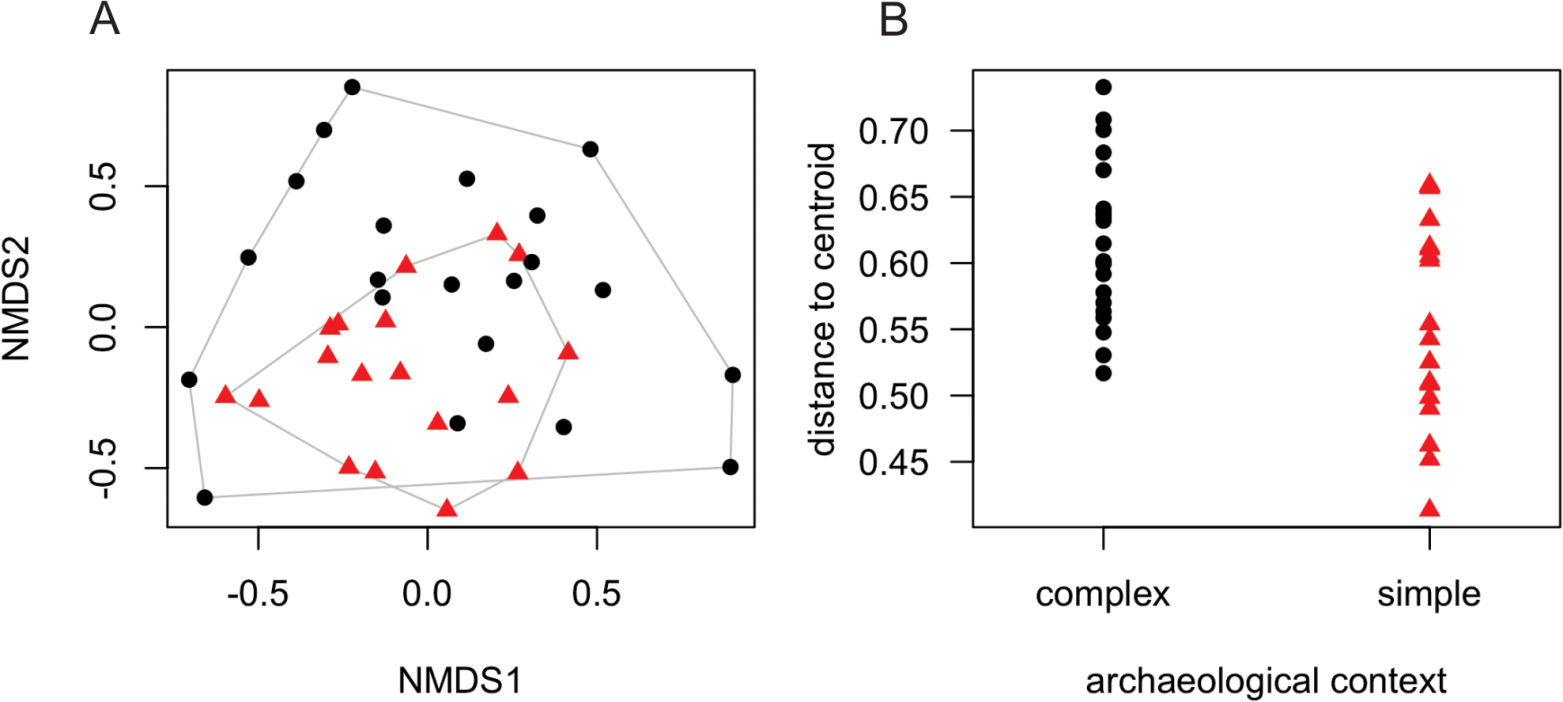
β-diversity of useful native Amazonian species in five communities along the Urubu River, Central Amazonia, Brazil. (A) Jaccard dissimilarities between homegarden floristic composition for each archaeological context (triangles = simple archaeological contexts; circles = complex archaeological contexts). The two NMDS axes accounted for 39% of the total floristic composition variation. The closer the points are the more similar floristic compositions are between the homegardens that the points represent. (B) Each triangle or circle represents the distance of one homegarden’s floristic composition in multivariate space to the centroid homegarden of the archaeological context. The floristic variability of homegarden sites in complex (multi-occupation) archaeological contexts is significantly different from that in simple (single-occupation) contexts (p = 0.009).

Homegarden floristic dissimilarity was not significantly different between archaeological contexts when all useful species were included (p = 0.096). However, with just the 75 native Amazonian species (see S1 Table), the results were striking: the effect of archaeological context was highly significant (p = 0.009) in explaining the variability in floristic compositions (Fig 2). The mixed effects model explained 35% of the floristic variability in the data matrix, of which archaeological context explained 59%, while soil fertility explained 39%, locality explained 3% and homegarden size had no measurable effect. This result holds even in simple comparisons (not controlling for any confounding effects). This effect of archaeological context was obscured by the introduction of non-Amazonian species that have little or no relation with the indigenous past (80 Old World species and 50 non-Amazonian New World species).

**Fig 3:**
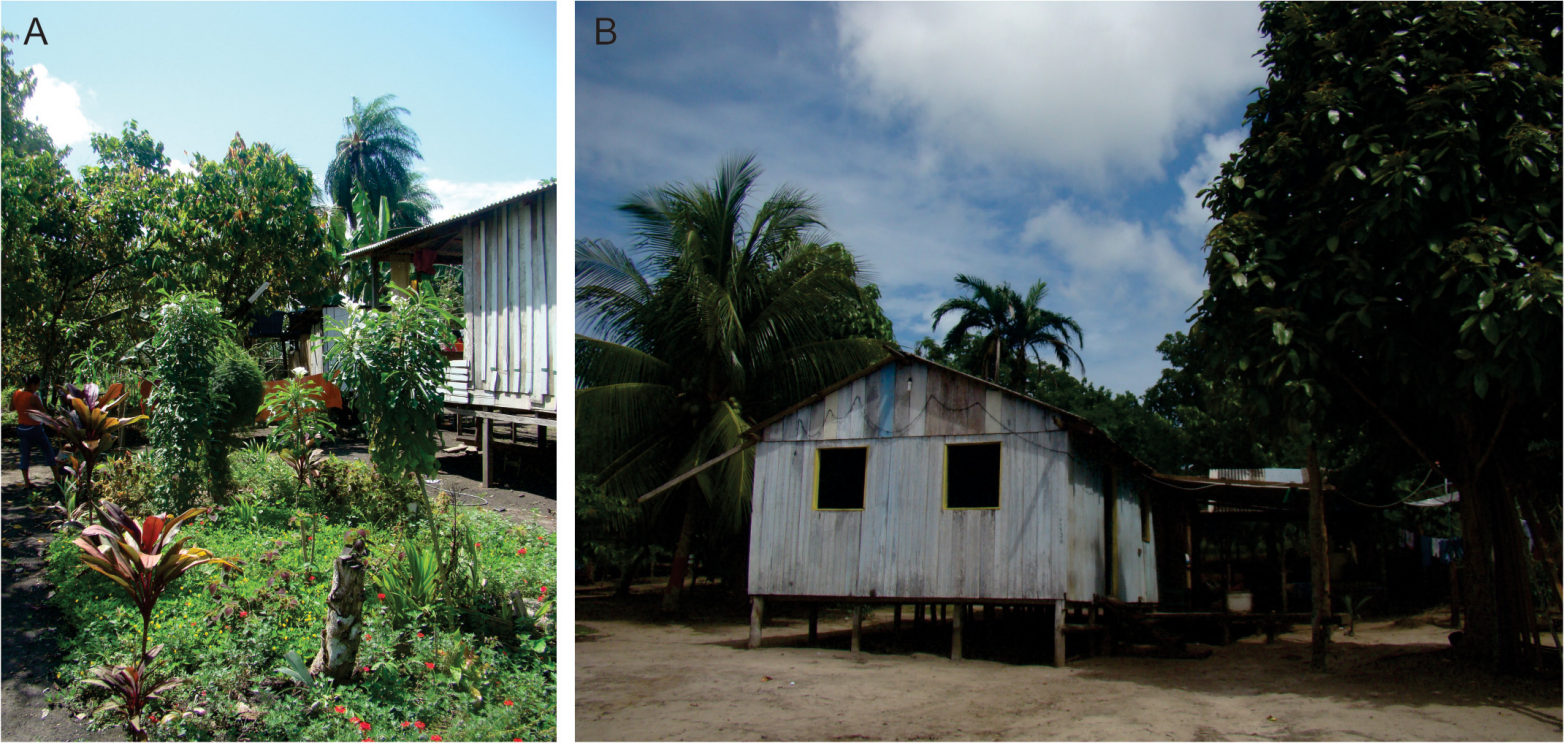
Homegardens located on *terra preta de índio* in the study region. (A) Homegarden in São José da Enseada with abundant α-diversity in all strata. (B) Homegarden in Irmandade Terra Preta with numerous fruit trees around (coconut tree, left; peach palm, center; malay apple, right). The herbaceous strata is behind this house. (Photos by Juliana Lins).

The average size of homegardens was 0.11±0.09 ha (Fig 3). The α-diversity of useful species within homegardens was high compared to other studies in Amazonia [23], with 205 species recorded in the 40 homegardens (excluding weeds). We recorded a mean of 26.3±15.2 species per homegarden, of which 8.2±6.6 native Amazonian species, 7.4±4.0 non-Amazonian New World species, and 10.7±5.6 Old World species. The most common native Amazonian species were cupuaçu (*Theobroma grandiflorum*), present in 65% of homegardens, sweet manioc (*Manihot esculenta*), present in 45%, and Amazonian hot pepper (*Capsicum chinense*), present in 43%. Hot pepper and manioc are crops with domesticated populations, and therefore totally dependent on human intervention; both were domesticated early and certainly were present in the region before European conquest [24], but probably represent reintroductions rather than legacies because they are herbaceous. Cupuaçu also may be a more recent introduction, arriving from eastern Pará State [25], although it was probably domesticated prior to European conquest [2].

The number of native Amazonian species in simple (50 species; mean = 7.32±6.68 per homegarden) and complex (61 species; mean = 9.00±6.66 per homegarden) archaeological contexts was similar (t-test, p = 0.43). However, the set of modern homegardens in complex archaeological contexts have more species combinations and are 10% more heterogeneous (β-diversity) than the set of homegardens in simple archaeological contexts (Fig 2). This means that the differences found in floristic compositions between the two archaeological contexts are due to combinations of species in the sets of homegardens (β-diversity) and not to the species richness of individual homegardens (α-diversity).

The higher β-diversity among modern homegardens established in complex archaeological contexts might reasonably be attributed to cultural variability through time, which is a legacy from the past. Different cultural groups are likely to have different preferences for food, fiber, medicinal and other useful species, and so are likely to have different useful species repertories [26]. Consequently, multiple occupations by different cultural groups through time may leave multiple legacies and the result is that greater cultural diversity in the past can lead to greater useful species diversity in the present in a given area.

Alternatively, the greater diversity found in complex archeological contexts could be explained by time since abandonment. Homegardens found in complex contexts were abandoned much more recently (perhaps 200–300 years prior to modern post-colonial reoccupation [27]) than the single-occupation contexts abandoned perhaps as much as a millennium ago [27]. Different species have different growth habits, life histories and degrees of domestication, and thus will disappear from domesticated landscapes at different rates after the abandonment [2, 28]. By the time the forest regenerated, after the collapse of the indigenous populations, the chances of greater survival in the seed banks of useful species that were present in past homegardens were higher in more recently abandoned sites. Most of these species were probably early successional species [15] and they may have been maintained in the seed bank by naturally occurring forest gaps through time. The longer the abandonment, the greater the chances of losing these light-demanding plants.

A third explanation for the legacy effect found in modern homegardens is modern traditional ecological knowledge. The species combinations in homegardens are strongly influenced by personal preferences [15] and it is quite common to find spontaneous useful plants in TPI [17]. Thus, it is logical to expect that current occupants take advantage of known elements left by past management, thus keeping and protecting desired species. The relative influence of these hypotheses, which are not mutually exclusive, can be tested with micro-archaeological analyses, such as pollen, phytoliths, starch grains and charcoal [29, 30].

### Conclusions

Our findings contribute to the debate about the magnitude and especially the durability of the ecological footprint of ancient indigenous occupations in Amazonia before and during European conquest [31]. We have shown that the legacies left by ancient human societies in Amazonia are found not only in the forest; they are also present in highly dynamic cultivated landscapes, suggesting that cultural diversity does contribute to biological diversity, as higher β-diversity was found in homegardens situated on archaeological sites with ceramics of different peoples. Also, our findings have implications for understanding the legacy of pre-Columbian landscape domestication. It had been already demonstrated that *terra preta de índio* contributes to agrobiodiversity conservation in second growth forests [32], which may once have been homegardens also. Now we show that these legacies can be identified in active homegardens, extremely dynamic agroecossystems that are continually shaped and re-shaped on an almost daily basis. Our results confirm and broaden the argument that it is not possible to understand current useful biodiversity in Amazonia and elsewhere without understanding past human activities [10].

## Material and Methods

### Study area

The survey was conducted in four riverside communities and a rural neighborhood in the municipalities of Silves and Itapiranga, located along the lower Urubu River, Amazonas, Brazil (Fig 1): Pontão (2° 49' 59.30'' S, 58° 11' 29.77'' W), Mucajatuba (2° 50' 33.21'' S, 58° 12' 29.64'' W), Irmandade Terra Preta (2° 48' 42.97'' S, 58° 13' 19.66'' W), São José da Enseada (2° 46' 03.04'' S, 58° 4' 8.91'' W) and Taperebatuba (2° 50' 15.43'' S, 58° 19' 17.84'' W). All localities have patches of TPI ranging from less than 1 ha to 10 ha [33, 34] in non-flooded areas. The natural vegetation of the area is dense evergreen rain forest, with periodically flooded forests along the floodplains.

The Urubu River has been known to Europeans since the seventeenth century due to its natural resources and the large number of indigenous villages along the river and its tributaries [35]. The river became famous for the massacres committed against indigenous people by the colonizers, with the first recorded conflict in 1663 [36]; these massacres gave the river its name—*urubu* means vulture in Portuguese and these were attracted in enormous numbers by the slaughter. According to Simões [35], who made the first systematic archaeological surveys of the region, the river remained long ignored and feared, serving as a hideout for runaway slaves and criminals until the late eighteenth century. There are dozens of archaeological sites along the river that may have either a single or more than one pre-Columbian occupation [27]. Currently there are dozens of non-indigenous communities with traditional peoples inhabiting mostly the bluffs along the river, most of which have TPI.

### Design and archaeological context

Altogether 40 homegardens were studied in 5 communities. The choice of homegardens took into account the presence of TPI in the homegarden, and informants suggested other informants—Snow Ball method [37], who were then asked to participate.

**Fig 4:**
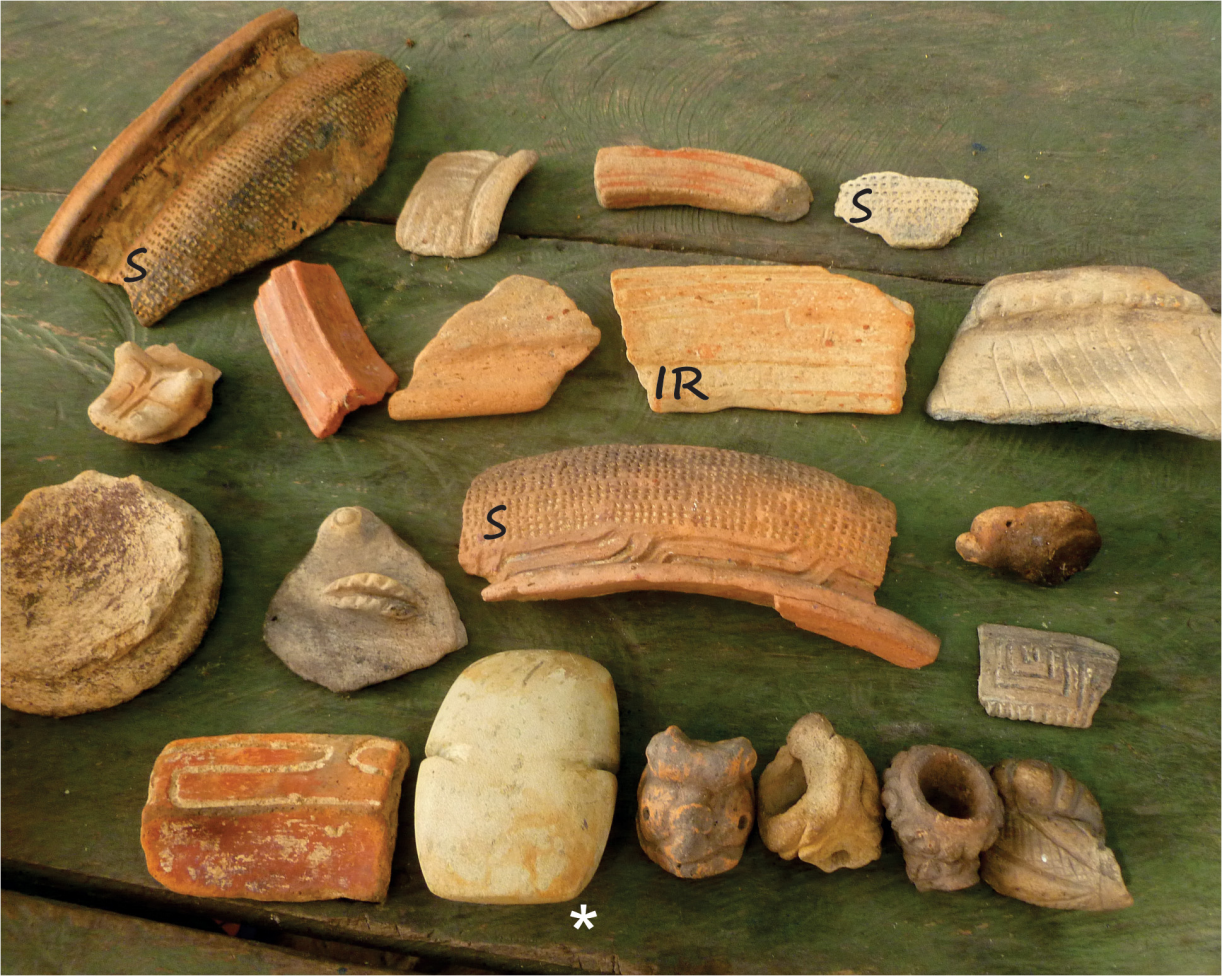
Personal archaeological collection of a community resident of Irmandade Terra Preta. This community is located on a multi-occupational archaeological site. The ceramic fragment identified as **IR** is a diagnostic fragment from the Incised Rim tradition and the ceramic fragments identified as **S** are diagnostic fragments from the Saracá tradition. It is normal to have greater representation of the latter ceramic fragments in surface collections as the oldest fragments are buried more deeply. The stone ax indicated by * is 10 cm in length. (Photo by Juliana Lins).

Two sites (Pontão and Mucajatuba) contained a single pre-Columbian occupation characterized by the Incised Rim [38] ceramic tradition from the 6^th^ and 9^th^ centuries AD [27], representing the earliest occupations in the area. Three sites (São José da Enseada, Irmandade Terra Preta and Taperebatuba) had complex occupational histories that correspond to at least two stratigraphically distinct occupations: the oldest is Incised Rim and the more superficial one is locally known as Saracá [39] (Fig 4), an occupation that continued until European conquest [27]. Local Incised Rim periods were radiocarbon dated [27] and Saracá were chronologically inferred by comparison with other dated sites in this region [27]. The Incised Rim occupations are usually attributed to Arawak speakers [40], who are famous for their agriculture. However it is not yet possible to relate Saracá ceramics to a particular linguistic group, although Tupi is likely [40]. There is no documentary information about reoccupation of the studied localities. From interviews with older residents, we believe that this happened about 100–150 years ago. In addition to botanical inventories, standard drivers of homegarden floristic diversity were also analyzed: soil composition (P, K, Ca, Mg, Fe, Zn and Mn concentrations), homegarden area and locality.

### Data Analysis

We measured the area of each homegarden as defined by the owner and carried out a floristic survey. Most plants in homegardens are well known, so each species was photographed and its common name was requested from the owner. The uncommon plants were collected and deposited in the Instituto Federal de Educação, Ciência e Tecnologia do Amazonas herbarium (EAFM), Manaus. The identifications were made by pictures, common names and voucher specimens. Native species identifications followed the Species List of Brazilian Flora [41] and exotic species followed The Plant List [42]. All species were categorized in terms of their origin for data analysis, and the categories were: native Amazonian, non-Amazonian New World and Old World. We also collected a sample of soil from the 0–20 cm horizon in each homegarden and analyzed pH in H_2_O, P, K, Ca, Mg, Fe, Zn, Mn. The analyses were performed at the Thematic Laboratory of Soils and Plants at INPA, using the EMBRAPA protocol [43].

For the analysis, each homegarden was treated independently because its management depends on the personal preferences and decisions of its owner. Principal Components Analysis (PCA) summarized the main soil nutrients in homegardens; the first PCA axis of soil characteristics was used in all subsequent analysis. We used the average distance to the group centroid (i.e., multivariate dispersion) as a measure of overall species diversity, or β-diversity, for each archaeological context [44], using the Jaccard index of presence/absence of floristic composition. One homegarden did not have any Amazonian species and another one had no non-Amazonian New World species and both were eliminated from the analysis. We created a 2 dimensional NMDS solution to present the differences in useful species heterogeneity between simple and complex archeological contexts. The NMDS analysis was also based on the Jaccard index of presence/absence of plant species native to Amazonia. Mixed effects models [22] were used to analyze our nested spatial design (homegardens within communities): the archaeological context, the first PCA axis of soil characteristics and homegarden area were fixed components, while locality was set as a random factor. Markov chain Monte Carlo-estimated p-values are presented for each factor in the analysis. All analyses were done in R [45] using vegan [46], lme4 [47] and languageR packages [48]. R scripts and input files used in the analyses are provided in Supporting Information (S1 Dataset), which also contains floristic and soil data for each homegarden.

### Ethics Statement

Due to the involvement of traditional communities, the project was approved by the Human Research Ethics Committee (CEP) at the National Institute of Amazonian Research (INPA), protocol number 024/11. As required by CEP-INPA, each person interviewed signed a prior and informed consent form that recognizes the intellectual property rights of the community members about the information provided, guarantees confidentiality to the informants, reaffirms that participation is voluntary, and explains the goals and methods of research.

## Supporting Information

S1 Dataset R scripts and input files used in the analyses. (RAR)

S1 Table Cultivated species found in 40 homegardens in two different archaeological contexts at 5 communities along the Urubu River, Amazonas, Brazil, in 2011. **All were categorized in terms of their origin: native Amazonian (n = 75), non-Amazonian New World (n = 50) and Old World (n = 80) species.** Abbreviations: Rf = relative frequency, HG = homegardens, AC = archaeological context.
(DOCX)
