# Supplementary material for: Pre-Columbian Floristic Legacies in Modern Homegardens of Central Amazonia
Source: PLoS One. 2015 Jun 1;10(6):e0127067. doi: 10.1371/journal.pone.0127067 (PMC4451503; doi:10.1371/journal.pone.0127067)
Supplement: S1 Table — All were categorized in terms of their origin: native Amazonian (n = 75), non-Amazonian New World (n = 50) and Old World (n = 80) species. Abbreviations: Rf = relative frequency, HG = homegardens, AC = archaeological context. (DOCX) [file pone.0127067.s002.docx]

**Table 2. Cultivated species found in 40 homegardens in two different archaeological contexts at 5 communities along the Urubu River, Amazonas, Brazil, in 2011. All were categorized in terms of their origin: native Amazonian (n=75), non-Amazonian New World (n=50) and Old World (n=80) species**.

| **family** | **Latin name** | **local common name** | **frequency (n/40)** | **Rf in simple AC HG** | **Rf in complex AC HG** |
| --- | --- | --- | --- | --- | --- |
|  | **Native Amazonian species (n=75)** |  |  |  |  |
|  |  |  |  |  |  |
| Acanthaceae | *Justicia calycina* (Nees) V.A.W.Graham | sara-tudo | 8 | 0,11 | 0,29 |
|  | *Justicia pectoralis* Jacq. | trevo-cumaru | 3 | 0,05 | 0,10 |
|  | *Justicia pectoralis* Jacq. forma mutuquinha | mutuquinha | 2 | 0,05 | 0,05 |
| Amaranthaceae | *Alternanthera brasiliana* (L.) Kuntze | roxa | 1 | 0,05 | 0,00 |
|  | *Alternanthera* sp. | cuia-mansa | 1 | 0,05 | 0,00 |
| Amaryllidaceae | *Crinum erubescens* L.f. ex Aiton | sucena | 3 | 0,05 | 0,10 |
| Annonaceae | *Annona montana* Macfad. | araticum | 1 | 0,05 | 0,00 |
|  | *Annona mucosa* Jacq. | biribá | 7 | 0,05 | 0,29 |
| Apiaceae | *Eryngium foetidum* L. | chicória | 15 | 0,53 | 0,24 |
| Apocynaceae | *Allamanda cathartica* L. | ─ | 2 | 0,05 | 0,05 |
|  | *Thevetia peruviana* (Pers.) K.Schum. | castanha-da-índia | 2 | 0 | 0,10 |
| Araceae | *Dieffenbachia* sp. | aninga, tajá | 5 | 0,21 | 0,05 |
|  | Unidentifed Araceae "tajá" | tajá | 1 | 0,05 | 0,00 |
|  | Unidentifed Araceae 1 | ─ | 1 | 0,05 | 0,00 |
|  | Unidentifed Araceae 2 | ─ | 1 | 0,05 | 0,00 |
|  | *Philodendron* sp. | tajá | 1 | 0 | 0,05 |
|  | *Syngonium* sp. | cachorrinho | 2 | 0,05 | 0,05 |
| Arecaceae | *Acrocomia aculeata* (Jacq.) Lodd. ex Mart. | mucajá | 2 | 0,11 | 0,00 |
|  | *Bactris gasipaes* Kunth | pupunha | 7 | 0,11 | 0,24 |
|  | *Euterpe oleracea* Mart. | açaí, açaí-vermelho | 7 | 0,16 | 0,19 |
|  | Unidentifed Arecaceae | ─ | 1 | 0 | 0,05 |
|  | *Oenocarpus bacaba* Mart. | bacaba | 9 | 0,16 | 0,29 |
| Aristolochiaceae | *Aristolochia* sp. | oecá | 1 | 0 | 0,05 |
| Asteraceae | *Acmella oleracea* (L.) R.K. Jansen | jambú | 6 | 0,16 | 0,14 |
|  | *Ayapana triplinervis* (M.Vahl) R.M.King & H.Rob. | japana, japana-branca | 2 | 0 | 0,10 |
| Bignoniaceae | *Fridericia chica* (Bonpl.) L.G.Lohmann | carajirú, crajiru | 8 | 0,16 | 0,24 |
|  | *Mansoa alliacea* (Lam.) A.H.Gentry | cipó-alho | 6 | 0,11 | 0,19 |
| Bixaceae | *Bixa orellana* L. | urucu, urucum | 11 | 0,16 | 0,38 |
| Cactaceae | *Brasiliopuntia brasiliensis*(Willd.) A.Berger | dinheiro | 1 | 0 | 0,05 |
| Caryocaraceae | *Caryocar villosum* (Aubl.) Pers. | pequiá | 1 | 0 | 0,05 |
| Chrysobalanaceae | *Couepia* cf. *subcordata* Benth. ex Hook.f. | mari | 2 | 0,05 | 0,05 |
| Clusiaceae | *Platonia insignis* Mart. | bacuri | 1 | 0 | 0,05 |
| Commelinaceae | Unidentifed Commelinaceae | ─ | 2 | 0 | 0,10 |
| Convolvulaceae | *Ipomoea quamoclit* L. | primavera, esqueleto | 1 | 0 | 0,05 |
| Costaceae | *Costus* sp. | pobre-velho | 3 | 0,05 | 0,10 |
| Cyperaceae | Unidentifed Cyperaceae "manufa" | manufa | 1 | 0 | 0,05 |
| Dioscoreaceae | *Dioscorea* sp. | cará-de-índio | 1 | 0 | 0,05 |
| Euphorbiaceae | *Croton sacaquinha* Croizat | sacaquinha | 3 | 0,05 | 0,10 |
|  | *Euphorbia tithymaloides* L. | corama, coramina, jiboia | 6 | 0,21 | 0,10 |
|  | *Hevea brasiliensis* (Willd. ex A.Juss.) Müll.Arg. | seringa | 2 | 0,05 | 0,05 |
|  | *Manihot esculenta* Crantz | macaxeira | 18 | 0,37 | 0,52 |
| Fabaceae | *Inga edulis* Mart. | ingá-de-metro, ingá-cipó | 8 | 0,11 | 0,29 |
|  | *Inga longiflora* Spruce ex Benth. | ingá | 4 | 0,05 | 0,14 |
|  | *Inga marginata* Willd. | ingá | 1 | 0 | 0,05 |
|  | Inga sp. | ingá | 13 | 0,47 | 0,19 |
|  | *Arachis repens* Handro | amendoim-forrageiro | 1 | 0 | 0,05 |
|  | *Cassia leiandra* Benth. | mari-mari | 3 | 0,05 | 0,10 |
| Gesneriaceae | *Episcia cupreata* (Hook.) Hanst. | ─ | 1 | 0,05 | 0,00 |
| Iridaceae | *Eleutherine bulbosa* (Mill.) Urb. | japiinzinho, bacabaí | 3 | 0,05 | 0,10 |
| Lamiaceae | *Ocimum campechianum* Mill. | favaquinha | 11 | 0,16 | 0,38 |
|  | *Scutellaria agrestis*  St.-Hil. ex Benth. | trevo-roxo | 8 | 0,21 | 0,19 |
| Malpighiaceae | *Bunchosia armeniaca* (Cav.) DC. | marmelo | 1 | 0,05 | 0,00 |
| Malvaceae | *Theobroma cacao* L. | cacau | 8 | 0,11 | 0,29 |
|  | *Theobroma grandiflorum* (Willd. ex Spreng.) K. Schum. | cupú, cupuaçu | 26 | 0,84 | 0,48 |
|  | *Theobroma speciosum* Willd. ex Spreng. | cacaurana | 1 | 0 | 0,05 |
| Marantaceae | *Calathea ornata* (Linden) Körn. | ─ | 1 | 0,05 | 0,00 |
|  | Unidentifed Marantaceae "vai-e-vem" | vai-e-vem | 1 | 0,05 | 0,00 |
|  | *Maranta* sp. | jandicá | 1 | 0,05 | 0,00 |
| Meliaceae | *Carapa guianensis* Aubl. | andiroba | 1 | 0 | 0,05 |
| Myrtaceae | *Eugenia uniflora* L. | pitanga | 3 | 0 | 0,14 |
| Phytolaccaceae | *Petiveria alliacea* L. | mucura-caá | 13 | 0,32 | 0,33 |
| Piperaceae | *Piper marginatum* Jacq. | caapema, caapiá | 1 | 0 | 0,05 |
|  | *Piper* sp. | pau-de-angola | 1 | 0 | 0,05 |
| Portulacaceae | *Portulaca pilosa* L. | amor-crescido | 2 | 0,11 | 0,00 |
| Pteridaceae | *Doryopteris* sp. | vence-tudo | 1 | 0 | 0,05 |
|  | *Pteris cretica* L. | samambaia | 1 | 0 | 0,05 |
| Sapindaceae | *Talisia cupularis* Radlk. | pitomba | 11 | 0,26 | 0,29 |
| Sapotaceae | *Pouteria caimito* (Ruiz & Pav.) Radlk. | abiu | 11 | 0,21 | 0,33 |
| Simaroubaceae | *Quassia amara* L. | quina-quina | 1 | 0 | 0,05 |
| Solanaceae | *Capsicum chinense* Jacq. | pimenta-cheirosa | 17 | 0,47 | 0,38 |
| Unidentified | Unidentifed "samadaru" | samadaru | 1 | 0 | 0,05 |
| Unidentified | Unidentifed "pata-de-boi" | pata-de-boi | 1 | 0 | 0,05 |
| Unidentified | Unidentifed "sangue-suga" | sangue-suga | 1 | 0,05 | 0,00 |
| Urticaceae | *Pilea microphylla* (L.) Liebm. | brilhantina | 1 | 0 | 0,05 |
| Verbenaceae | *Lippia alba* (Mill.) N.E.Br. ex Britton & P.Wilson | erva-cidreira | 11 | 0,21 | 0,33 |
|  |  |  |  |  |  |
|  | **non-Amazonian New World species (n=50)** |  |  |  |  |
|  |  |  |  |  |  |
| Acanthaceae | *Pachystachys lutea* Nees | camarão | 1 | 0 | 0,05 |
| Amaranthaceae | *Chenopodium ambrosioides* L. | mastruz | 5 | 0,11 | 0,14 |
| Anacardiaceae | *Anacardium occidentale* L. | caju | 15 | 0,26 | 0,48 |
|  | *Spondias mombin* L. | taperebá | 3 | 0,05 | 0,10 |
| Annonaceae | *Annona muricata* L. | graviola | 13 | 0,21 | 0,43 |
|  | *Annona squamosa* L. | ata | 5 | 0,16 | 0,10 |
| Apocynaceae | *Plumeria pudica* Jacq. | buquê-de-noiva | 2 | 0 | 0,10 |
|  | *Plumeria rubra* L. | ─ | 1 | 0,05 | 0,00 |
| Araceae | *Anthurium andraeanum* Linden ex André | ─ | 1 | 0,05 | 0,00 |
| Arecaceae | *Mauritia flexuosa* L.f. | buriti | 6 | 0,11 | 0,19 |
|  | *Oenocarpus bataua* Mart. | patauá | 1 | 0,05 | 0,00 |
| Asparagaceae | *Agave angustifolia* Haw. | ─ | 1 | 0 | 0,05 |
|  | *Agave attenuata* Salm-Dyck | ─ | 1 | 0 | 0,05 |
|  | *Agave* cf. *sisalana* Perrine | ─ | 1 | 0 | 0,05 |
|  | *Yucca filamentosa* L. | ─ | 1 | 0,05 | 0,00 |
| Asteraceae | *Pluchea sagittalis* Less. | macela | 3 | 0,11 | 0,05 |
|  | *Tagetes erecta* L. | cravo-amarelo | 1 | 0 | 0,05 |
| Bignoniaceae | *Crescentia cujete* L. | cuia | 8 | 0,21 | 0,19 |
| Bromeliaceae | *Ananas comosus* (L.) Merr. | abacaxi | 16 | 0,32 | 0,48 |
| Cactaceae | *Cereus jamacaru* DC. | ─ | 1 | 0,05 | 0,00 |
|  | *Opuntia ficus-indica* (L.) Mill. | palma, palmatória | 4 | 0,05 | 0,14 |
| Campanulaceae | *Hippobroma longiflora* (L.) G.Don | ─ | 1 | 0 | 0,05 |
| Caricaceae | *Carica papaya* L. | mamão | 15 | 0,32 | 0,43 |
| Chrysobalanaceae | *Licania tomentosa* (Benth.) Fritsch | ─ | 1 | 0,05 | 0,00 |
| Commelinaceae | *Tradescantia pallida* (Rose) D.R.Hunt | boca-de-lobo | 2 | 0 | 0,10 |
|  | *Tradescantia spathacea* Sw. | ─ | 1 | 0 | 0,05 |
| Convolvulaceae | *Ipomoea batatas* (L.) Poir. | batata-doce | 1 | 0 | 0,05 |
| Cucurbitaceae | *Cucurbita* sp. | jerimum | 8 | 0,32 | 0,10 |
| Euphorbiaceae | *Jatropha curcas* L. | pinhão-branco | 11 | 0,26 | 0,29 |
|  | *Jatropha gossypiifolia* L. | pinhão-roxo | 18 | 0,53 | 0,38 |
|  | *Jatropha podagrica* Hook. | pinhão-barrigudo | 6 | 0 | 0,29 |
| Fabaceae | *Libidibia ferrea* (Mart. ex Tul.) L.P.Queiroz | jucá | 5 | 0,05 | 0,19 |
| Lauraceae | *Persea americana* Mill. | abacate | 16 | 0,42 | 0,38 |
| Lythraceae | *Cuphea gracilis*Kunth | ─ | 6 | 0,11 | 0,19 |
| Malpighiaceae | *Malpighia emarginata* DC. | acerola | 14 | 0,32 | 0,38 |
| Malvaceae | *Gossypium barbadense* L. | algodão | 8 | 0,16 | 0,24 |
| Myrtaceae | *Psidium guajava* L. | goiaba | 25 | 0,58 | 0,67 |
| Nyctaginaceae | *Bougainvillea spectabilis* Willd. | "roseira" | 4 | 0,16 | 0,05 |
| Passifloraceae | *Passiflora edulis* Sims | maracujá, peroba | 14 | 0,32 | 0,38 |
| Piperaceae | *Peperomia argyreia* (Hook.f.) E.Morren | irapuru | 1 | 0 | 0,05 |
| Portulacaceae | *Portulaca* cf. *grandiflora* Hook. | onze-horas | 2 | 0 | 0,10 |
| Rubiaceae | *Genipa americana* L. | jenipapo | 5 | 0,05 | 0,19 |
| Sapotaceae | *Manilkara zapota* (L.) P.Royen | sapotilha | 1 | 0 | 0,05 |
| Solanaceae | *Brunfelsia uniflora* (Pohl) D.Don | ─ | 1 | 0 | 0,05 |
|  | *Capsicum annuum* L. | pimentão | 7 | 0,05 | 0,29 |
|  | *Capsicum annuum* var. *glabriusculum* (Dunal) Heiser & Pickersgill | pimenta-de-mesa | 1 | 0 | 0,05 |
|  | *Capsicum frutescens* L. | pimenta-malagueta | 16 | 0,47 | 0,33 |
|  | *Cestrum nocturnum* L. | ─ | 1 | 0 | 0,05 |
|  | *Solanum lycopersicum* L. | tomate | 8 | 0,05 | 0,33 |
| Verbenaceae | *Lippia microphylla* Cham. | salvinha | 6 | 0,05 | 0,24 |
|  |  |  |  |  |  |
|  | **Old World species (n=80)** |  |  |  |  |
|  |  |  |  |  |  |
| Acanthaceae | *Asystasia gangetica* (L.) T.Anderson | ─ | 1 | 0 | 0,05 |
|  | *Crossandra nilotica* Oliv. | ─ | 1 | 0 | 0,05 |
|  | *Thunbergia erecta* (Benth.) T.Anderson | ─ | 1 | 0 | 0,05 |
| Amaranthaceae | *Celosia argentea*L. | crista-de-galo | 3 | 0,05 | 0,10 |
| Amaryllidaceae | *Allium fistulosum* L. | cebolinha, cebola | 19 | 0,32 | 0,62 |
|  | *Scadoxus* sp. | coroa-de-nossa-senhora | 1 | 0 | 0,05 |
| Anacardiaceae | *Mangifera indica* L. | manga | 30 | 0,84 | 0,67 |
| Apiaceae | *Coriandrum sativum* L. | coentro | 5 | 0,05 | 0,19 |
| Apocynaceae | *Catharanthus roseus* (L.) G. Don | lavandeira, rosa-branca | 8 | 0,16 | 0,24 |
|  | *Tabernaemontana divaricata* (L.) R.Br. ex Roem. & Schult. | rosa-branca | 3 | 0,05 | 0,10 |
| Araceae | *Alocasia* × *mortfontanensis* André | ─ | 1 | 0,05 | 0,00 |
| Araliaceae | *Polyscias cumingiana* (C.Presl) Fern.-Vill. | sabugo | 1 | 0,05 | 0,00 |
|  | *Polyscias guilfoylei* (W.Bull) L.H.Bailey | taperebazinho | 4 | 0,16 | 0,05 |
| Arecaceae | *Cocos nucifera* L. | coco | 28 | 0,84 | 0,57 |
| Asparagaceae | *Cordyline fruticosa*(L.) A.Chev. | ─ | 7 | 0,32 | 0,05 |
| Asparagaceae | *Dracaena braunii* Engl. | ─ | 1 | 0 | 0,05 |
|  | *Dracaena godseffiana* hort. | jiboinha | 1 | 0,05 | 0,00 |
|  | *Sansevieria trifasciata* Prain | jiboia | 5 | 0,16 | 0,10 |
| Asteraceae | *Gymnanthemum amygdalinum* (Delile) Sch.Bip. ex Walp. | boldo | 1 | 0,05 | 0,00 |
| Balsaminaceae | *Impatiens balsamina* L. | ─ | 1 | 0 | 0,05 |
| Brassicaceae | *Brassica oleracea* var. *viridis* L. | couve | 6 | 0,11 | 0,19 |
| Crassulaceae | *Bryophyllum daigremontianum* (Raym.-Hamet & Perrier) A.Berger | ─ | 1 | 0,05 | 0,00 |
|  | *Bryophyllum pinnatum* (Lam.) Oken | corama, pirarucu-caá | 7 | 0,21 | 0,14 |
| Cucurbitaceae | *Cucumis anguria* L. | maxixe | 3 | 0,05 | 0,10 |
|  | *Trichosanthes cucumerina* L. | quiabo-de-metro | 1 | 0 | 0,05 |
|  | *Citrullus lanatus* (Thunb.) Matsum. & Nakai | melancia | 2 | 0 | 0,10 |
| Dioscoreaceae | *Dioscorea alata* L. | cará-de-mingau | 1 | 0 | 0,05 |
|  | *Dioscorea bulbifera* L. | cará-do-ar | 1 | 0,05 | 0,00 |
| Euphorbiaceae | *Acalypha wilkesiana* Müll.Arg. | ─ | 2 | 0,05 | 0,05 |
|  | *Codiaeum variegatum* (L.) Rumph. ex A.Juss. | brasileirinha | 7 | 0,11 | 0,24 |
|  | *Euphorbia milii*Des Moul. | coroa-de-cristo | 1 | 0 | 0,05 |
|  | *Euphorbia tirucalli* L. | cachorro-pelado | 1 | 0,05 | 0,00 |
| Fabaceae | *Chamaecrista desvauxii* aff. var. *latistipula* (Benth.) G.P.Lewis | ─ | 1 | 0 | 0,05 |
|  | *Erythrina variegata* L. | ─ | 1 | 0 | 0,05 |
|  | *Tamarindus indica* L. | tamarino | 1 | 0 | 0,05 |
| Iridaceae | Unidentifed "mão-do-diabo" | mão-do-diabo | 1 | 0 | 0,05 |
|  | *Neomarica* sp. | ─ | 1 | 0 | 0,05 |
| Lamiaceae | *Aeollanthus suaveolens* Mart. ex Spreng. | catinga-de-mulata | 1 | 0 | 0,05 |
|  | *Mentha* cf. *spicata* L. | vique, hortelãzinho | 4 | 0,05 | 0,14 |
|  | *Ocimum americanum* L. | manjericão | 2 | 0 | 0,10 |
|  | *Ocimum basilicum* L. | alfavaca-cheirosa | 3 | 0,05 | 0,10 |
|  | *Ocimum gratissimum* L. | alfavaca, alfavacão | 9 | 0,21 | 0,24 |
|  | *Plectranthus amboinicus* (Lour.) Spreng. | hortelã-grande | 7 | 0,21 | 0,14 |
|  | *Plectranthus neochilus* Schltr. | boldo-do-miúdo | 6 | 0,16 | 0,14 |
|  | *Plectranthus scutellarioides* (L.) R.Br. | ─ | 3 | 0 | 0,14 |
|  | *Pogostemon heyneanus* Benth. | oriza | 1 | 0,05 | 0,00 |
|  | *Vitex agnus-castus* L. | ─ | 1 | 0 | 0,05 |
| Lauraceae | *Cinnamomum verum* J.Presl | canela | 1 | 0 | 0,05 |
| Lythraceae | *Lagerstroemia indica* L. | loucura | 6 | 0,11 | 0,19 |
| Malvaceae | *Hibiscus rosa-sinensis* L. | matizada, papoula | 13 | 0,26 | 0,38 |
| Moraceae | *Artocarpus altilis* (Parkinson ex F.A.Zorn) Fosberg | fruta-pão | 1 | 0,05 | 0,00 |
|  | *Artocarpus heterophyllus* Lam. | jaca | 2 | 0,05 | 0,05 |
|  | *Ficus benjamina* L. | ─ | 1 | 0 | 0,05 |
| Musaceae | *Musa* × *paradisiaca* L. | banana | 32 | 0,79 | 0,81 |
| Myrtaceae | *Syzygium cumini* (L.) Skeels | azeitona | 9 | 0,16 | 0,29 |
|  | *Syzygium malaccense* (L.) Merr. & L.M.Perry | jambo | 15 | 0,32 | 0,43 |
| Oleaceae | *Jasminum multiflorum* (Burm.f.) Andrews | buquê-de-noiva | 2 | 0 | 0,10 |
| Oxalidaceae | *Averrhoa bilimbi* L. | limão-caiana | 2 | 0,05 | 0,05 |
|  | *Averrhoa carambola* L. | carambola | 4 | 0,05 | 0,14 |
| Pedaliaceae | *Sesamum indicum* L. | gergelim | 1 | 0,05 | 0,00 |
| Phyllanthaceae | *Breynia disticha* J.R.Forst. & G.Forst. | ─ | 1 | 0,05 | 0,00 |
| Piperaceae | *Piper nigrum* L. | pimenta-do-reino | 1 | 0 | 0,05 |
| Poaceae | *Cymbopogon citratus* (DC) Stapf. | capim-cheiroso | 12 | 0,26 | 0,33 |
|  | *Saccharum officinarum* L. | cana | 7 | 0,16 | 0,19 |
| Rosaceae | *Rosa* × *hybrida* Schleich. | rosa | 2 | 0,05 | 0,05 |
| Rubiaceae | *Coffea arabica* L. | café, café-bandinha | 8 | 0,11 | 0,29 |
|  | *Coffea canephora* Pierre ex A.Froehner | café-moca, café-mococa | 8 | 0,11 | 0,29 |
|  | *Ixora coccinea* L. | ─ | 9 | 0,05 | 0,38 |
|  | *Morinda citrifolia* L. | noni | 2 | 0,05 | 0,05 |
|  | *Mussaenda erythrophylla* Schumach. & Thonn. | ─ | 1 | 0 | 0,05 |
| Rutaceae | *Citrus* ×*limon* (L.) Osbeck. | limão-tangerina | 1 | 0 | 0,05 |
|  | *Citrus aurantiifolia* (Christm.) Swingle | lima | 3 | 0 | 0,14 |
|  | *Citrus* cf. *limon* (L.) Burm. f. | limão | 21 | 0,53 | 0,52 |
|  | *Citrus reticulata* Blanco | tangerina | 8 | 0,21 | 0,19 |
|  | *Citrus sinensis* (L.) Osbeck | laranja | 33 | 0,84 | 0,81 |
|  | *Ruta graveolens* L. | arruda | 6 | 0,05 | 0,24 |
| Verbenaceae | *Verbena* sp. | ─ | 2 | 0 | 0,10 |
| Xanthorrhoeaceae | *Aloe vera* (L.) Burm.f. | babosa | 6 | 0,16 | 0,14 |
| Zingiberaceae | *Zingiber officinale* Roscoe | mangarataia | 8 | 0,16 | 0,24 |
|  | *Zingiber zerumbet* (L.) Roscoe ex Sm. | mangarataia-tauá | 2 | 0,05 | 0,05 |

Abbreviations: Rf = relative frequency, HG = homegardens, AC = archaeological context.
